# A nomogram for predicting intraoperative risk during primary percutaneous coronary intervention based on rapidly obtained data from ST-segment elevation myocardial infarction patients

**DOI:** 10.3389/fcvm.2026.1691709

**Published:** 2026-02-12

**Authors:** Yutian Wang, Xiao Chen, Yejia Chen, Chen Yu, Yiming Tao, Xiaocong Liu, Kaile Zhao, Haoran Shou, Hua Liu, Jiancheng Xiu, Xiaobo Li

**Affiliations:** 1 Guangdong Provincial Key Laboratory of Cardiac Function and Microcirculation, Department of Cardiology, Nanfang Hospital, Southern Medical University, Guangzhou, Guangdong, China; 2 Department of Cardiology, The First Affiliated Hospital of Nanchang University, Nanchang, Jiangxi, China; 3 Department of Biostatistics, School of Public Health, Southern Medical University, Guangzhou, Guangdong, China; 4 The Xiangya School of Medicine, Central South University, Changsha, China; 5 Department of Cardiology, Xiangdong Hospital Hunan Normal University, Liling City, Hunan, China

**Keywords:** cardiogenic shock, malignant arrhythmias, nomogram prediction model, no-reflow, PCI, STEMI

## Abstract

**Background:**

Primary percutaneous coronary intervention (PCI) in patients with ST-segment elevation myocardial infarction (STEMI) can carry high stakes, as major adverse cardiac events (MACE) like no-reflow, malignant arrhythmias, and cardiogenic shock can disrupt the procedure and worsen outcomes. In light of these challenges, this study aimed to develop a nomogram prediction model to rapidly assess MACE risk during PCI in STEMI patients, aiding in timely risk stratification prior to surgery.

**Methods:**

This study included 1050 STEMI patients who underwent primary PCI between December 30, 2016, and May 13, 2023. Clinical data were collected from emergency admissions. Multiple logistic regression models were used to analyze the independent risk factors for intraoperative MACE. A nomogram was then constructed and validated via bootstrap resampling. Model performance was assessed using an ROC curve for discrimination and a calibration curve for accuracy.

**Results:**

The incidence of intraoperative MACE in STEMI patients was 38.3%. Independent risk factors for intraoperative MACE included Killip classification, ST-segment elevation in ≥3 leads, white blood cell count, lymphocyte count, and heart rate. A simple and rapidly obtainable nomogram, developed to predict MACE during PCI, showed good differentiation, with an area under the ROC curve of 0.785 (95% CI: 0.755–0.814). Decision curve analysis indicated good fit, calibration, and positive net benefits.

**Conclusions:**

A nomogram was developed to rapidly predict intraoperative MACE risk during PCI in STEMI patients before surgery. By enabling early identification of high-risk individuals, this model enhances clinical decision-making, potentially improving patient outcomes and procedural efficiency.

## Introduction

Acute ST-segment elevation myocardial infarction (STEMI) is a common life-threatening condition in industrialized nations, with its incidence and mortality increasing at unprecedented rates in some low- and middle-income countries. Percutaneous coronary intervention (PCI) is the preferred treatment for STEMI, as it effectively restores blood flow in occluded vessels and improves myocardial perfusion (1). Timely reperfusion is crucial for preserving myocardial tissue and improving patient prognosis.

Although the promotion of chest pain centers has optimized preprocedural workflows, major adverse cardiovascular events (MACE), such as cardiogenic shock, malignant arrhythmias (ventricular tachycardia, ventricular fibrillation, cardiac arrest, etc.), and other adverse events may occur during PCI. After recanalization of occluded coronary arteries, the no-reflow phenomenon, reperfusion arrhythmia, and other complications may also occur in acute myocardial infarction (AMI) patients (2). Studies have shown that approximately 10%–30% of patients who undergo primary PCI may experience no-reflow after recanalization of occluded vessels (1), and approximately 4.3% may experience ventricular tachycardia and ventricular fibrillation during PCI (3). There are few studies on the risk factors for cardiogenic shock during PCI. Intraoperative MACE poses a direct risk to prognosis and prolongs the procedure, delaying reperfusion and further worsening patient outcomes (4–11). Preventing and managing these intraoperative events to further shorten reperfusion times may become a future focus for optimizing chest pain centers.

Currently, measures for preventing and treating MACE during primary PCI are lacking. Early identification of high-risk patients and timely preparations can help doctors implement reasonable interventions before surgery, improve the operation success rate, reduce the operative time, and lower the risk of postoperative MACE.

Prediction models for no-reflow risk in STEMI patients after PCI have been based on biochemical and clinical characteristics (12). Wongthida developed a scoring system using related indicators to predict life-threatening arrhythmias in STEMI patients after PCI (13). The establishment of these risk prediction models has played a positive role in preventing the risk of primary PCI. However, some parameters in these models, such as liver and kidney function indicators, require at least 90 min to measure. In clinical practice, the need for a door-to-balloon time under 90 min limits the usefulness of prediction models requiring long data acquisition times. Additionally, these models only evaluate no-reflow and arrhythmia risks and lack relevant assessments for intraoperative cardiogenic shock. Moreover, prediction models specifically designed to assess composite intraoperative MACE in patients with STEMI undergoing primary PCI remain relatively limited. All the risk events mentioned above can prolong the operation time, increase the operation risk, and affect the prognosis.

Therefore, the main objective of this study was to establish a convenient and stable nomogram prediction model for MACE during primary PCI in STEMI patients, based on clinical and laboratory parameters that are routinely and rapidly obtainable in emergency settings. These readily available parameters include blood test results, electrocardiogram (ECG) findings, and basic medical history obtained at or near the time of first medical contact. Furthermore, this study aimed to evaluate the predictive value of this model for in-hospital outcomes in STEMI patients undergoing primary PCI. This model aims to provide a reliable tool for the early prediction and prevention of MACE in high-risk STEMI patients during primary PCI. This will facilitate a comprehensive assessment of operational risks, enhance preoperative discussions with patients' families, and improve overall preparation, ultimately leading to better patient outcomes.

## Methods

### Patients and grouping

A total of 1,050 STEMI patients who underwent primary PCI at Xiangdong Hospital Affiliated to Hunan Normal University between December 30, 2016, and May 13, 2023, were initially selected. The following patients were excluded: (1) those who died during PCI; (2) those who received thrombolytic therapy within 12 h of onset; (3) those with severe mechanical complications; and (4) those without recorded blood pressure or with a systolic blood pressure less than 90 mmHg. After specific patients were excluded, 998 patients (759 males and 239 females) were included in the study. We have illustrated the flow of participants through the study in a flowchart (Supplementary Figure S1). On the basis of the occurrence of MACE, the 998 patients were divided into two groups: 383 patients who experienced MACE and 615 patients who did not. The incidence of MACE was 38.3%. The research protocol adheres to the ethical guidelines of the 1975 Declaration of Helsinki. Approval for this study was obtained from the Ethics Committee of Xiangdong Hospital, Hunan Normal University (No. 2021002). As a retrospective study, patient-related indicators were collected for statistical analysis without additional interventions. The risks to the subjects were solely those associated with conventional treatment and not related to this study. The primary risk in this study was the protection of privacy. Therefore, all identifiable information about patients was removed from the study materials. Written informed consent was waived for this clinical study.

### Collection of clinical data

The data collected included general clinical information, intraoperative PCI data, and preoperative laboratory results that that are routinely obtainable at the time of first medical contact in the emergency department or at the very early stage of primary PCI. These data were recorded in chest pain forms, electronic medical record systems, and the Chinese Chest Pain Center data reporting platform.

The general clinical information included sex, age, transfer from another hospital, medication treatment at the referring hospital, ST-segment elevation in ≥3 leads, hypertension, hyperlipidemia, diabetes, smoking history, Killip classification at admission, time from chest pain onset to PCI, occurrence of cardiac arrest before PCI, preoperative antihypertensive medication, heart rate at admission, and systolic and diastolic blood pressure. Killip classification was assessed at admission by experienced physicians based on patients’ initial clinical presentation and physical examination.

The laboratory tests included high-sensitivity troponin, hemoglobin, red blood cell count, white blood cell count, neutrophil count, platelet count, lymphocyte count, blood glucose, mean platelet volume, serum sodium, serum potassium, and fibrinogen. All laboratory samples were collected at the time of first medical contact and processed through the emergency laboratory system according to standard clinical practice.

Intraoperative PCI data included events of cardiogenic shock and malignant arrhythmias during PCI, a thrombus shadow on coronary angiography, identification of diseased vessels, the number of stents used, the use of an intra-aortic balloon pump (IABP), and preoperative intravenous antihypertensive medication.

### Definition of various MACE during primary PCI

All the patients with STEMI who underwent primary PCI received dual antiplatelet therapy consisting of aspirin (300 mg loading dose) and a P2Y12 receptor inhibitor (ticagrelor 180 mg or clopidogrel 600 mg) before or at the initiation of the procedure. Two experts performed coronary angiography via the Judkins technique and tailored the PCI procedure on the basis of the offending vessel's condition. Intraoperative MACE, including no-reflow, malignant arrhythmias, and cardiogenic shock, were defined by procedure delays and the need for drug or device interventions. Acute heart failure was initially considered as a potential component of the composite endpoint but was ultimately excluded due to difficulty in establishing a clear and consistent intraoperative diagnosis.

No-reflow**:** After PCI, two experts with over five years of primary PCI experience assessed coronary flow status based on angiographic findings using the thrombolysis in myocardial infarction (TIMI) flow-grading system. TIMI grades 0, 1, and 2 are defined as no-reflow, whereas TIMI grade 3 is defined as reflow. If there was a disagreement between the two experts, a third expert with more than ten years of primary PCI experience was consulted.

Malignant Arrhythmias: These are arrhythmias during PCI that cause hemodynamic instability, potentially leading to syncope or death. These arrhythmias include ventricular fibrillation, ventricular tachycardia, third-degree atrioventricular block, and sick sinus syndrome.

Cardiogenic Shock: Defined as severe acute peripheral circulatory failure due to significantly reduced cardiac function, cardiogenic shock is identified by a drop in systolic blood pressure below 90 mmHg during the operation in patients who have normal blood pressure preoperatively and are not on antihypertensive drugs.

Adverse intraoperative events were identified based on the need for urgent pharmacological or device-based interventions during the procedure. No-reflow was defined by the requirement for intracoronary vasodilators, such as nicorandil or sodium nitroprusside. Malignant arrhythmias were identified by the administration of antiarrhythmic agents (e.g., amiodarone, lidocaine, or atropine) and/or the use of emergency devices, including temporary pacemakers or defibrillators. Cardiogenic shock was identified by the requirement for vasoactive agents (including metaraminol or dopamine) and/or mechanical circulatory support, such as IABP, during the procedure.

### Statistical analysis

#### Missing data handling and data preparation

Missing values were handled prior to model development using multiple imputation. Imputation methods were automatically specified according to variable type: predictive mean matching (PMM) for continuous variables, logistic regression for binary variables, and multinomial logistic regression for categorical variables. Multiple imputation was performed using the random forest–based *missRanger* algorithm, which is capable of capturing complex nonlinear relationships among variables. The imputed dataset was then used for subsequent analyses. Before modeling, the cohort was randomly divided into a training set and a validation set at a ratio of 7:3.

#### Descriptive statistics and group comparisons

The Kolmogorov–Smirnov test was used to determine whether continuous variables conformed to a normal distribution. Variables that did conform are expressed as the mean ± standard deviation, whereas those that did not conform are expressed as the median [interquartile range, i.e., 25th−75th percentile (IQR)]. Categorical variables are expressed as numbers or percentages. Differences among the groups were identified via Student's t test, the Mann–Whitney *U*-test, and the chi-square test. Spearman correlation coefficients were used for correlation analysis.

#### Variable selection using LASSO regression

To identify key predictors associated with intraoperative MACE and to reduce the risk of overfitting, least absolute shrinkage and selection operator (LASSO) regression was applied in the training set. All candidate predictors were standardized before analysis. Ten-fold cross-validation was used to determine the optimal regularization parameter (λ). The λ value corresponding to one standard error of the minimum deviance (lambda.1se) was selected, and variables with non-zero coefficients at this *λ* were retained for subsequent model construction.

#### Final model construction using multivariable logistic regression

Variables selected by LASSO regression were subsequently entered into a standard multivariable logistic regression model to estimate adjusted effect sizes. Regression coefficients (β) were estimated using maximum likelihood methods, and results are presented as odds ratios (ORs) with corresponding 95% confidence intervals (CIs).

#### Model evaluation and validation

The multicollinearity of the variables was tested via the variance inflation factor (VIF), with a VIF >10 indicating multicollinearity. After the total score for each patient was calculated via the nomogram risk prediction model, receiver operating characteristic (ROC) curve analysis was performed to determine the discrimination ability of the nomogram model. The Hosmer–Lemeshow goodness-of-fit test was used to determine the agreement between the probability that the nomogram model predicted MACE during primary PCI and the actual probability. Internal bootstrap validation was used with repeated sampling (1,000 repetitions) to verify the accuracy of the nomogram model. Decision curve analysis (DCA) was used to evaluate the clinical validity of the nomogram model.

#### Statistical software and significance level

All tests were two-tailed. Statistical analyses were conducted via R, version 4.2.3 (R Foundation for Statistical Computing). Differences with *P* < 0.05 were considered statistically significant.

## Results

### Clinical characteristics

A total of 998 participants were included in the study, with 383 patients experiencing MACE and 615 patients not experiencing MACE. Compared with the non-MACE group, patients in the MACE group were more likely to be female and had lower systolic and diastolic blood pressures on admission. They also had a higher Killip classification, longer time from chest pain onset to PCI, and a higher incidence of pre-PCI cardiac arrest.

With regard to laboratory parameters, the MACE group had higher white blood cell count, neutrophil count, platelet count, blood glucose levels, serum potassium levels, and high-sensitivity troponin, while lymphocyte counts were significantly lower. In addition, patients with MACE more frequently presented with ST-segment elevation in ≥3 leads, visible thrombus on coronary angiography, higher rates of transfer from another hospital, greater use of prehospital medications, and more frequent use of intra-aortic balloon pump (IABP). Detailed baseline characteristics are shown in Table 1.

**Table 1:** Baseline characteristics of participants who did and did not experience intraoperative MACE during PCI.

Characteristic		No-Mace	Mace	Statistics	*P*-Value
		*N* = 615	*N* = 383		
Age, Median (Q1, Q3)		64.0 [55.0, 71.0]	65.0 [56.0, 71.5]	Z = −1.096	0.273
Sex (%)	Female	127 (20.7)	112 (29.2)	*Χ*^2^ = 16.160	0.003
	Male	488 (79.3)	271 (70.8)		
Systolic Blood Pressure, Median (Q1, Q3)		133.0 [120.0, 150.0]	126.0 [110.0, 146.0]	Z = −3.865	<0.001
Diastolic Blood Pressure, Median (Q1, Q3)		80.0 [70.0, 90.0]	78.0 [70.0, 88.5]	Z = −3.513	<0.001
Red Blood Cell Count, Median (Q1, Q3)		4.5 [4.1, 4.9]	4.4 [4.0, 4.8]	Z = −2.244	0.025
Hemoglobin, Median (Q1, Q3)		138.0 [126.0, 148.0]	136.0 [122.0, 146.0]	Z = −1.849	0.064
White Blood Cell Count, Median (Q1, Q3)		9.4 [7.6, 11.5]	11.9 [9.5, 14.6]	Z = −10.570	<0.001
Mean Platelet Volume, Median (Q1, Q3)		10.6 [9.9, 11.3]	10.5 [9.8, 11.3]	Z = −1.166	0.244
Neutrophil Count, Median (Q1, Q3)		6.6 [4.9, 8.7]	9.7 [7.5, 12.2]	Z = −12.871	<0.001
Platelet Count, Median (Q1, Q3)		208.0 [173.0, 246.0]	225.0 [190.0, 273.0]	Z = −4.952	<0.001
Lymphocyte Count, Median (Q1, Q3)		1.7 [1.2, 2.5]	1.3 [1.0, 1.8]	Z = −6.972	<0.001
Heart Rate, Median (Q1, Q3), (beats/min)		75.0 [65.5, 87.0]	75.0 [65.0, 90.0]	Z = −0.320	0.749
High-sensitivity Troponin, Median (Q1, Q3)		0.4 [0.0, 3.3]	0.9 [0.1, 8.3]	Z = −3.605	<0.001
Time from Chest Pain to PCI (minutes), Median (Q1, Q3)		146.0 [74.0, 359.0]	199.0 [113.5, 411.0]	Z = −4.285	<0.001
Blood Glucose, Median (Q1, Q3)		7.1 [5.7, 9.4]	7.5 [6.0, 10.5]	Z = −2.636	0.008
Fibrinogen, Median (Q1, Q3)		3.1 [2.7, 3.6]	3.2 [2.7, 3.7]	Z = −1.500	0.134
Na, Median (Q1, Q3)		141.7 [139.3, 144.0]	141.2 [138.8, 143.8]	Z = −1.878	0.06
K, Median (Q1, Q3)		3.9 [3.6, 4.2]	3.9 [3.7, 4.3]	Z = −2.178	0.029
Pulse Pressure, Median (Q1, Q3)		51.0 [41.0, 60.0]	50.0 [40.0, 60.0]	Z = −3.104	0.002
Smoking History, *n* (%)	No	381 (62.0)	256 (66.8)	*χ*^2^ = 3.613	0.135
	Yes	234 (38.0)	127 (33.2)		
Hypertension, *n* (%)	No	256 (41.6)	172 (44.9)	*χ*^2^ = 0.629	0.34
	Yes	359 (58.4)	211 (55.1)		
Diabetes, *n* (%)	No	448 (72.8)	285 (74.4)	*χ*^2^ = 0.014	0.637
	Yes	167 (27.2)	98 (25.6)		
Killip Classification, *n* (%)	1	508 (82.6)	252 (65.8)	*χ*^2^ = 86.883	<0.001
	2	84 (13.7)	58 (15.1)		
	3	23 (3.7)	73 (19.1)		
Hyperlipidemia, *n* (%)	No	534 (86.8)	341 (89.0)	*χ*^2^ = 0.622	0.352
	Yes	81 (13.2)	42 (11.0)		
Pre-PCI Cardiac Arrest, *n* (%)	0	607 (98.7)	364 (95.0)	*χ*^2^ = 14.393	0.001
	1	8 (1.3)	19 (5.0)		
Transferred from Another Hospital, *n* (%)	No	439 (71.4)	239 (62.4)	*χ*^2^ = 9.849	0.004
	Yes	176 (28.6)	144 (37.6)		
Prehospital Medication, *n* (%)	No	500 (81.3)	282 (73.6)	*χ*^2^ = 11.180	0.005
	Yes	115 (18.7)	101 (26.4)		
ST-segment Elevation ≥ 3 Leads, *n* (%)	<3	338 (55.0)	98 (25.6)	*χ*^2^ = 129.925	<0.001
	≥3	277 (45.0)	285 (74.4)		
Visible Thrombus on Coronary Angiography, *n* (%)	No	207 (33.7)	91 (23.8)	*χ*^2^ = 16.970	0.001
	Yes	408 (66.3)	292 (76.2)		
Number of Stents, *n* (%)	1	339 (55.1)	210 (54.8)	*χ*^2^ = 4.565	0.265
	2	209 (34.0)	126 (32.9)		
	3	52 (8.5)	33 (8.6)		
	4	14 (2.3)	9 (2.3)		
	5	1 (0.2)	5 (1.3)		
IABP, *n* (%)	No	604 (98.2)	350 (91.4)	*χ*^2^ = 37.926	<0.001
	Yes	11 (1.8)	33 (8.6)		

IABP, intra-aortic balloon pump.

The study population was randomly divided into a training set (*n* = 697) and a validation set (*n* = 301) at a 7:3 ratio. Baseline characteristics of patients with and without MACE in the training cohort showed similar patterns to those observed in the total cohort and are summarized in Supplementary Table S1.

### Clinical features of the training set and validation set

The model was constructed via the training set and validated with data from the validation set. The balance test results indicated that, except for the variable “medication treatment from other hospitals”, there were no statistically significant differences between the training and validation sets, indicating good randomization (Supplementary Table S2).

### Construction of the predictive model for intraoperative MACE in acute STEMI patients

**Figure 1:**
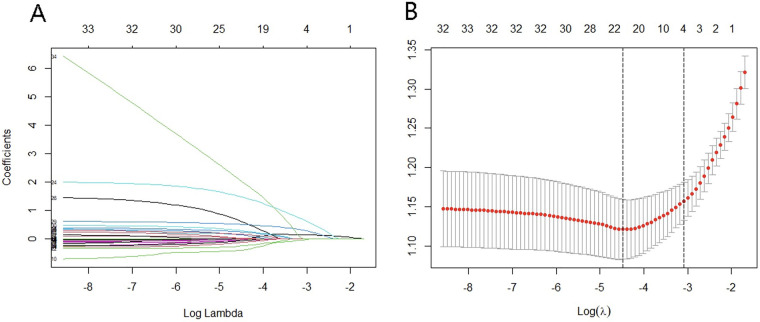
Variable screening based on LASSO regression. **(A)** Characteristics of changes in variable coefficients. **(B)** Selection process of optimal values of parameter *λ* in the LASSO regression model with the cross-validation method.

Feature selection was performed via the LASSO regression model. The optimal lambda parameter in the LASSO model was validated by plotting the relationship curve between the partial likelihood deviance (binomial deviance) and lambda (Figures 1A,B). A dashed vertical line was drawn based on the minimum standard (1-SE standard) at 1SE. The features with nonzero coefficients were selected by optimizing lambda. The results revealed that when the optimal lambda value was 0.0113, the independent factors associated with intraoperative MACE identified from the training cohort were white blood cell count, number of ST-segment elevation leads ≥3, lymphocyte count, Killip classification, and heart rate. The regression coefficients of the other indicators were compressed to zero and excluded.

### Development of personalized prediction models

The multicollinearity among the variables selected by LASSO regression was tested, and the results revealed that the variance inflation factors (VIFs) of the five selected variables were all less than 10. This finding indicates that there was no multicollinearity among the variables, allowing them to be included in the multivariable logistic analysis (Supplementary Table S3).

**Figure 2:**
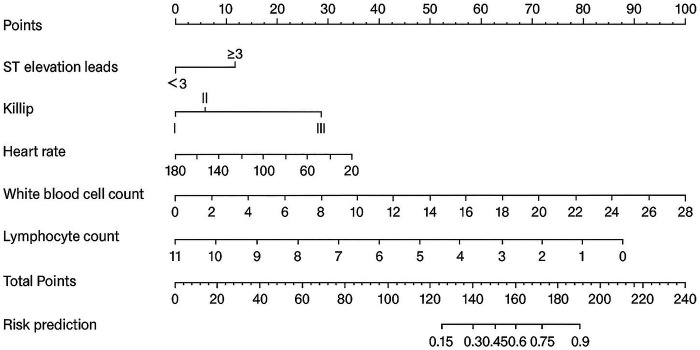
Nomogram of intraoperative adverse cardiovascular events.

On the basis of the predictive factors for intraoperative MACE identified through LASSO regression, logistic regression analysis was performed to construct a prediction model for the occurrence of intraoperative MACE in patients with AMI. The predictive factors included white blood cell count, number of ST-segment elevation leads ≥3, lymphocyte count, Killip classification, and heart rate. In multivariable logistic regression analysis, higher white blood cell count, a greater number of ST-segment elevation leads, higher Killip class, lower lymphocyte count, and lower heart rate were independently associated with an increased risk of intraoperative MACE (Supplementary Table S4). We further utilized the five significant risk factors to construct a nomogram (Figure 2). Each indicator corresponds to a score on the upper scoring line. The total score is the sum of the scores of these five indicators. This total score is then projected onto the bottom scale to indicate the probability of MACE occurring in AMI patients.

**Figure 3:**
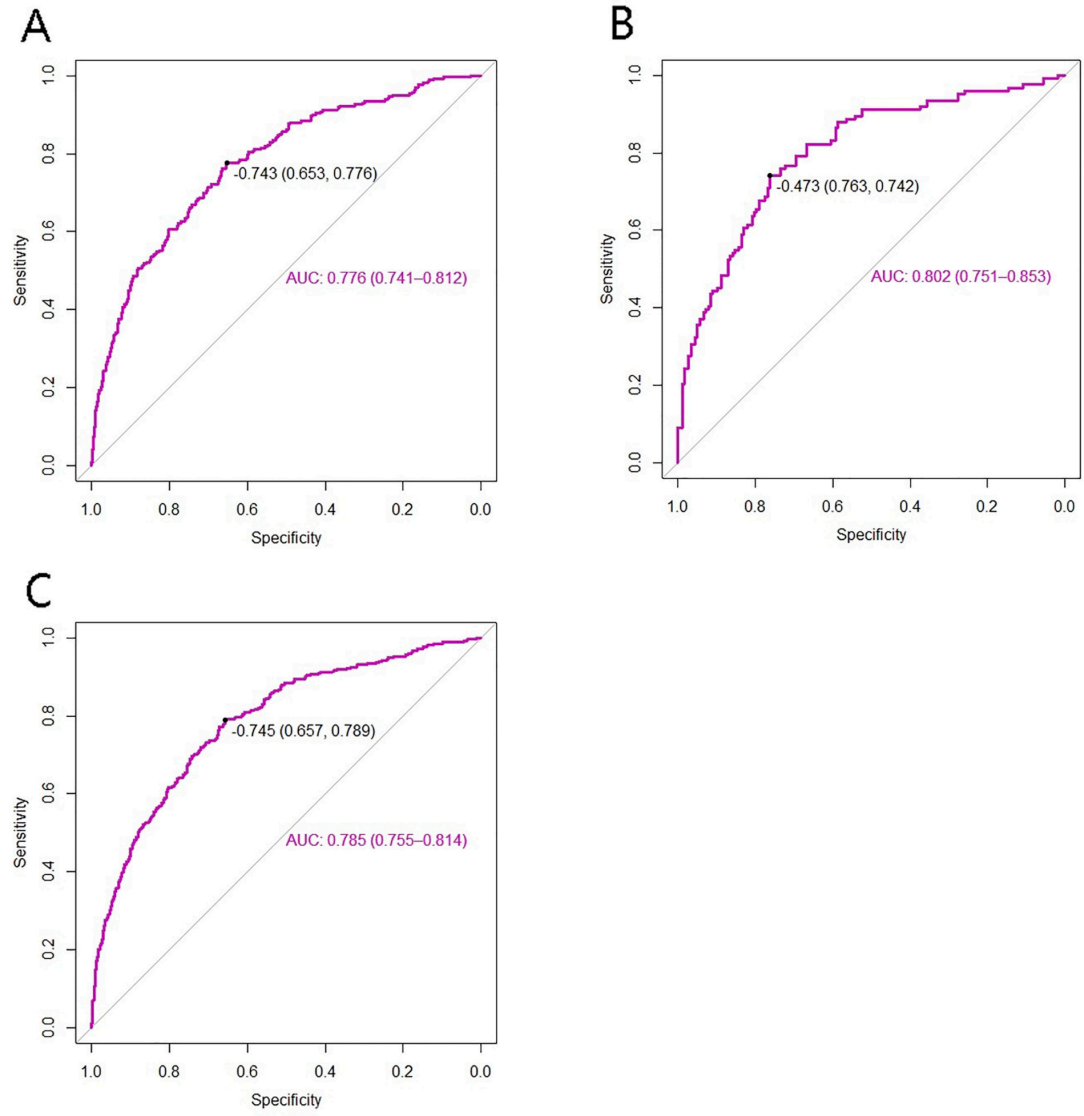
ROC curves of the model for intraoperative adverse cardiovascular events. **(A)** training set; **(B)** test set; **(C)** total set.

After the total score for each patient was calculated via the nomogram risk prediction model, ROC curve analysis was performed to evaluate the discriminatory ability of the model. The area under the ROC curve (AUC) for the training cohort was 0.776 (95% CI: 0.741–0.812). For the validation cohort, the AUC was 0.802 (95% CI: 0.751–0.853), with a specificity of 0.763 and a sensitivity of 0.742. In the total cohort, the AUC was 0.785 (95% CI: 0.755–0.814), with a specificity of 0.657 and a sensitivity of 0.789 (Figures 3A–C). In addition, the discriminatory performance of the established model was further evaluated for individual clinically relevant outcomes, including cardiogenic shock, malignant arrhythmias, and no-reflow. The model demonstrated acceptable discrimination across these individual outcomes, with detailed results presented in Table 2 and Supplementary Figure S2.

**Table 2:** Discriminatory performance of the established model for individual clinically relevant outcomes.

Outcome	AUC (95% CI)
Cardiogenic shock	0.770 (0.737–0.803)
Malignant arrhythmias	0.738 (0.690–0.787)
No-reflow	0.713 (0.670–0.756)

**Figure 4:**
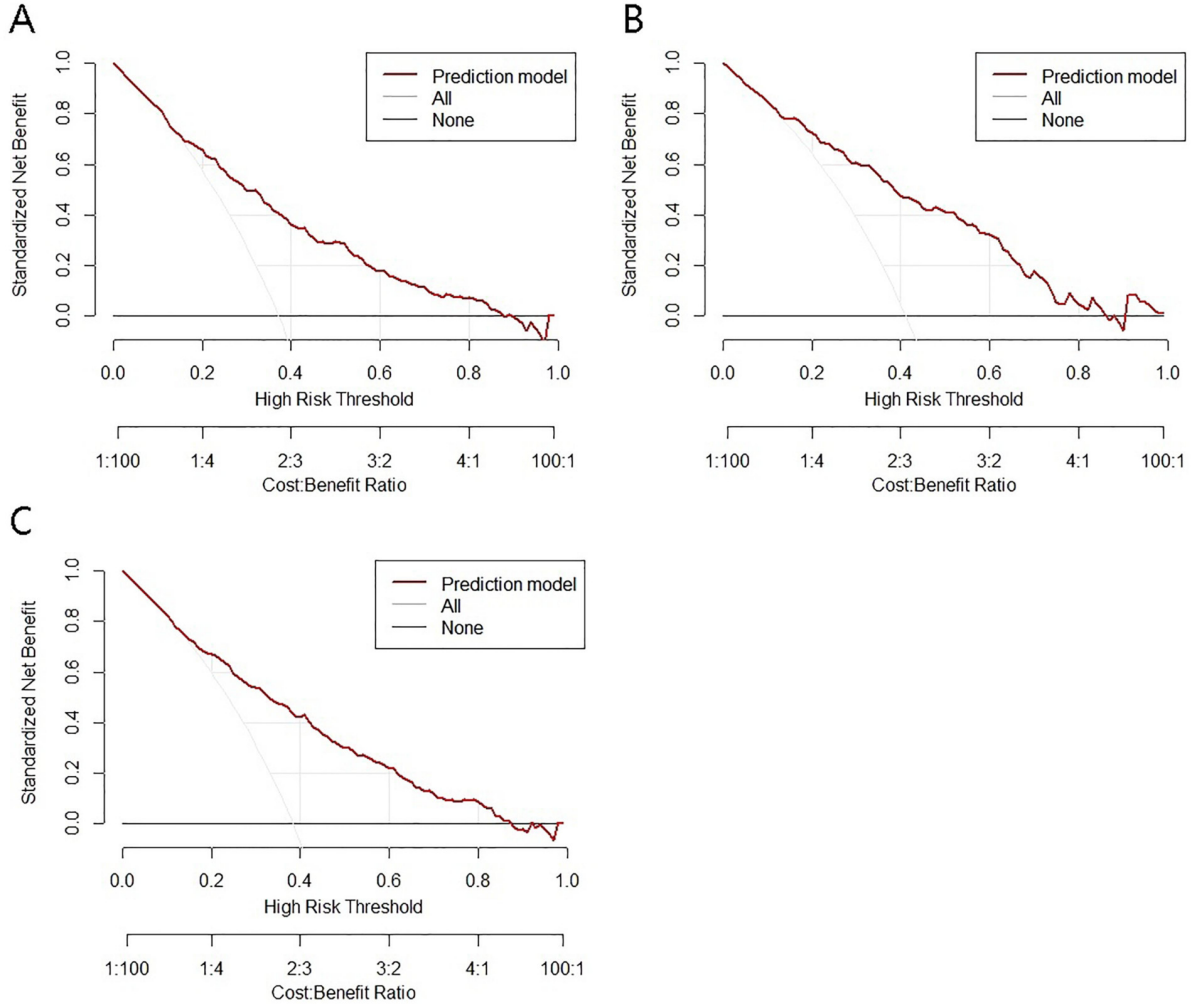
Decision curves of the model for intraoperative adverse cardiovascular events. **(A)** training set; **(B)** test set; **(C)** total set.

The decision curve analysis (DCA) for the nomogram model is illustrated in the corresponding figures. In the training cohort, the risk threshold for intraoperative MACE was 0.06–0.87; in the validation cohort, it was 0.04–0.86; and in the total cohort, it was 0.05–0.87. Within these ranges, the model demonstrated a positive net clinical benefit (Figures 4A–C).

**Figure 5:**
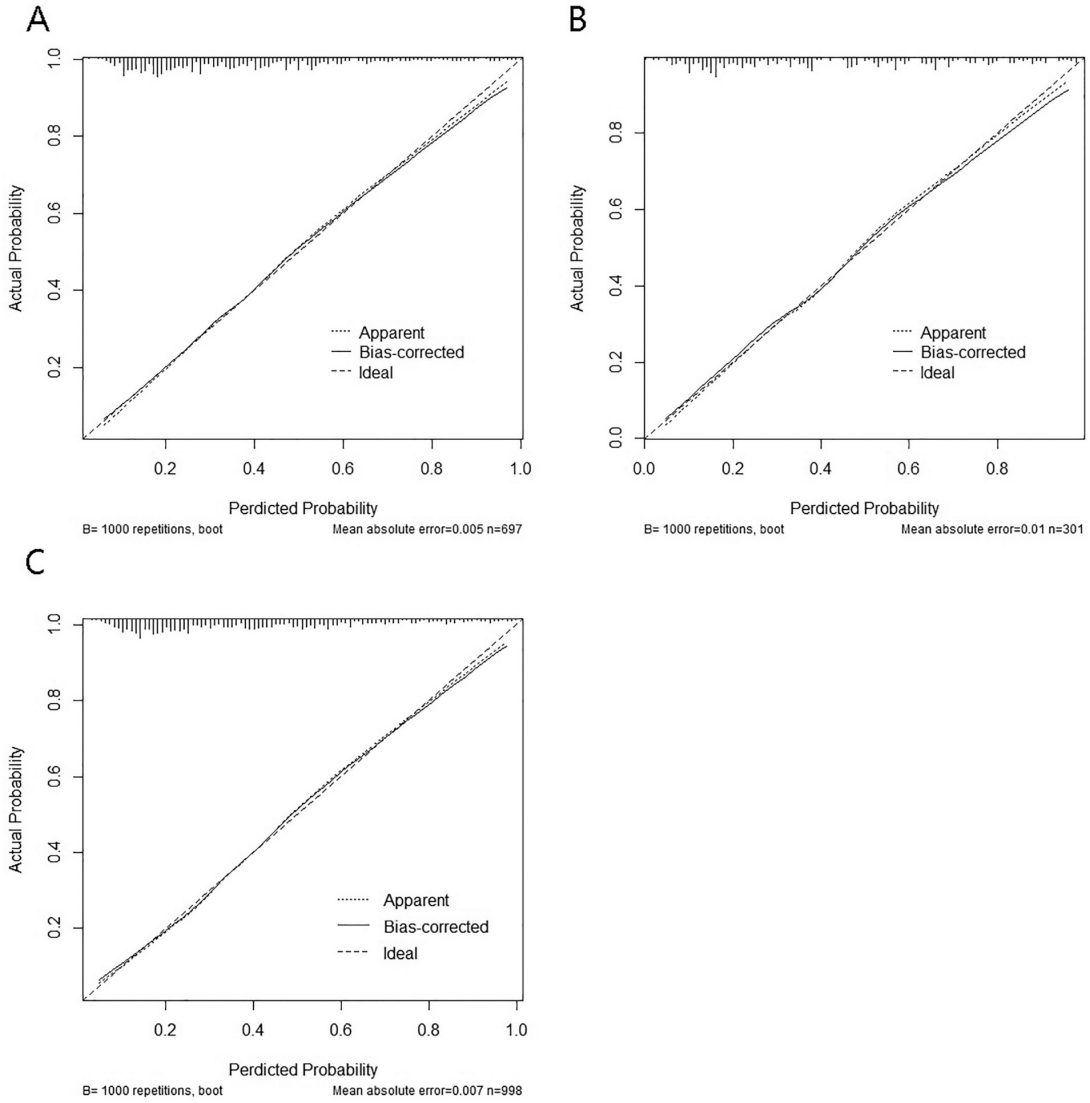
Calibration curves of the model for intraoperative adverse cardiovascular events. **(A)** training set; **(B)** test set; **(C)** total set.

The calibration curves indicated a high degree of overlap between the diagonal reference line and calibration curves, indicating good consistency between the predicted and actual probabilities of intraoperative MACE. The mean absolute error for the training cohort was 0.005, that for the validation cohort was 0.01, and that for the total cohort was 0.007, indicating good calibration across all cohorts (Figures 5A–C).

The goodness of fit of the nomogram model was further evaluated using the Hosmer–Lemeshow test. The results showed no significant lack of fit in either the training cohort (*χ*^2^ = 6.27, df = 8, *P* = 0.617) or the validation cohort (*χ*^2^ = 7.03, df = 8, *P* = 0.533), indicating good calibration of the model.

To accommodate the urgent nature of emergency surgery for acute STEMI, we designed an online calculator based on the nomogram developed in this study (available at lxb86.shinyapps.io/dn20240324/). This calculator can be used on both computer devices and various mobile terminals, allowing clinicians and surgeons to quickly and conveniently obtain real-time risk assessments for adverse cardiovascular events during primary PCI on the basis of patient clinical data (Supplementary Figure S3).

## Discussion

In this study, we first employed Lasso and multivariable logistic regression analyses to identify independent risk factors for intraoperative MACE during the first PCI in STEMI patients. In contrast to previous models that primarily target post-PCI or in-hospital outcomes or single complications, our model focuses on a composite intraoperative endpoint, which may better reflect real-world procedural risk during primary PCI. The identified indicators included white blood cell count, ST-segment elevation in ≥3 leads, lymphocyte count, Killip classification, and heart rate. On the basis of these risk factors, we constructed a nomogram risk prediction model for MACE during PCI, which demonstrated good predictive performance and clinical utility.

Several widely used risk scores in acute coronary syndrome (ACS) and PCI settings, such as the GRACE and TIMI risk scores, have demonstrated good prognostic value for post-PCI or in-hospital mortality and ischemic outcomes (14, 15). However, these tools were not designed to predict intraoperative complications occurring during primary PCI. First, their primary endpoints differ substantially from the composite intraoperative MACE evaluated in the present study, which includes malignant arrhythmias, cardiogenic shock, and no-reflow during the procedure itself. Second, these established scores are typically applied at admission or after PCI and are not intended for real-time procedural risk assessment. Third, some required variables in existing scores or previously published PCI-related nomogram models may not be routinely available before or at the very beginning of primary PCI in emergency settings. Therefore, rather than replacing established ACS/PCI risk scores, the present nomogram is intended to complement them by addressing a distinct clinical question focused on intraoperative risk stratification and procedural preparedness.

In the present study, decision curve analysis (DCA) was performed to evaluate the net clinical benefit of the nomogram across a range of threshold probabilities rather than to define a single mandatory intervention cutoff. The relatively wide threshold probability ranges observed in the training, validation, and total cohorts indicate that the model may provide positive net benefit across different risk tolerance levels in primary PCI settings.

Clinically, the nomogram is intended to support early intraoperative risk stratification and procedural preparedness rather than to dictate specific interventions at a fixed risk threshold. For patients identified as having a higher predicted risk of intraoperative MACE, operators may consider enhanced preparation at the beginning of PCI, such as readiness for advanced hemodynamic support, antiarrhythmic management, and prevention strategies for no-reflow, in accordance with institutional protocols and clinical judgment.

Although low density lipoprotein cholesterol (LDL-C) has been confirmed as an important causal risk factor for myocardial infarction, many patients with AMI do not have high LDL-C levels but exhibit increased inflammation. Residual inflammation is more common than residual high LDL-C in AMI patients (16). Inflammation plays a critical role in the occurrence and development of myocardial infarction, with neutrophils (the most abundant white blood cells in human blood) being the primary cells that appear in the damaged myocardium post-infarction, marking the most damaging phase of the inflammatory response (17). Monocytes and macrophages, which follow neutrophils into the myocardium, play key roles in post-infarction ventricular remodeling (18). These inflammatory cells are crucial in coronary atherosclerosis and thrombosis (19). Our findings suggest that a greater number of neutrophils and monocytes and a lower number of lymphocytes are associated with a greater risk of intraoperative MACE. Previous studies have confirmed that neutrophils and monocytes play important roles in atherosclerosis, thrombosis, myocardial injury, and ventricular remodeling post-myocardial infarction. The SII, NLR, and PLR have been shown to be important predictors of risk in STEMI patients undergoing PCI (20, 21). Our study further confirms the significant predictive value of inflammatory indices for intraoperative risk.

The number of leads with ST-segment elevation and the extent of ST-segment elevation are closely related to infarct size and patient prognosis (22). Quantifying the extent of the elevation of leads can be challenging, but the number of leads is a simple and easily obtainable data point. In this study, we found that as the number of leads with ST-segment elevation increased, the corresponding score in the nomogram model also increased. This finding indicates that a greater number of leads with ST-segment elevation correlates with greater intraoperative risk and poorer prognosis. Similarly, Killip classification is also closely related to patient prognosis. A higher Killip classification is associated with increased mortality in both STEMI and non-ST-elevation acute coronary syndrome patients (23). In a retrospective cross-sectional study conducted at Jinnah Hospital in Lahore, Pakistan, the in-hospital mortality rates for STEMI patients in Killip classes I to IV were 9.9%, 8.7%, 92.6%, and 100% (24), respectively. In our study, we excluded patients in Killip class IV because patients with preoperative cardiogenic shock often receive vasopressor medications to achieve a preoperative systolic blood pressure >90 mmHg and may use an IABP, leading to differences in preoperative blood pressure and blood pressure at the first medical contact, which may not reflect the patient's actual blood pressure status. Additionally, the high mortality rate in patients with cardiogenic shock means that they often receive sufficient attention from doctors, making the construction of a predictive model less meaningful. Studies have shown that the Killip classification is an independent risk factor for no-reflow direct PCI for STEMI patients and can predict in-hospital risk (25). Our study revealed that the Killip classification is an independent risk factor for intraoperative MACE in STEMI patients undergoing direct PCI.

Studies have shown that the use of beta-blockers within five hours of myocardial infarction does not reduce myocardial damage, improve myocardial remodeling, or decrease the incidence of early malignant arrhythmias (26). Furthermore, not all beta-blockers can mitigate myocardial reperfusion injury. The cardioprotective effect of metoprolol is mediated through a neutrophil target that other beta-blockers lack, rather than through heart rate control and sympathetic inhibition (27). While a high heart rate predicts poor long-term outcomes, bradycardia during the acute phase of STEMI increases the likelihood of intraoperative third-degree atrioventricular block, cardiac arrest, and sick sinus syndrome. Additionally, bradycardia may trigger bradycardia-tachycardia syndrome, leading to ventricular malignant arrhythmias (28). Bradycardia can reduce the effective circulating blood volume and is often a response of the vagus nerve during surgery, which can easily lead to cardiogenic shock. Our study revealed that bradycardia is a high-risk factor for intraoperative adverse cardiovascular events, providing a reference for the timing and type of beta-blocker use in chest pain center-related STEMI patients.

Cardiogenic shock, malignant arrhythmia (ventricular tachycardia, ventricular fibrillation, severe bradycardia), and no-reflow phenomenon may occur during primary PCI, and these events can significantly increase the risk of operation (2). Early identification of patients who may experience intraoperative MACE and the need for relevant preparations in advance can help facilitate timely management of adverse events, thus opening occluded blood vessels as soon as possible and minimizing procedural delays. For patients at risk of no-reflow, thrombus aspiration may be considered, or the number of predilation procedures can be reduced, with drugs such as adenosine, verapamil, and sodium nitroprusside prepared in advance for intracoronary administration (29). Patients at risk of malignant arrhythmias may benefit from the preparation of antiarrhythmic drugs (e.g., amiodarone, lidocaine), pacemakers, and defibrillators (30). For patients at risk of cardiogenic shock, vasopressors (e.g., dopamine, metaraminol), IABP, ECMO, and ventilators can be prepared according to institutional practice and operator judgment (31, 32). Therefore, early identification of high-risk patients who may experience adverse events during primary PCI will help doctors implement reasonable interventions before and during surgery, reduce the operation time, improve the operation success rate, and potentially reduce the risk of postoperative MACE.

## Study limitations

Despite these promising results, our study has several limitations. As a single-center, retrospective study with a relatively small sample size, selection bias may be present. Although our nomogram model demonstrated good stability and clinical benefit through internal bootstrap validation, external validation using a larger, multicenter cohort is needed to increase its generalizability. Future work will focus on expanding the sample size and conducting multicenter external validation to improve the model's stability and clinical applicability.

## Conclusions

In this study, we developed a nomogram risk prediction model for intraoperative MACE during primary PCI in STEMI patients, based on independent risk factors identified through Lasso and multivariable logistic regression analyses. The key predictors included white blood cell count, ST-segment elevation in ≥3 leads, lymphocyte count, Killip classification, and heart rate. The model demonstrated good predictive performance and clinical utility.

Our findings highlight the critical role of inflammation in intraoperative risk, as increased neutrophil and monocyte counts and decreased lymphocyte counts were associated with a higher risk of adverse events. Additionally, the number of leads with ST-segment elevation and Killip classification were strong predictors of intraoperative MACE. Bradycardia was identified as a high-risk factor, providing insights into the timing and selection of beta-blocker use in STEMI patients.

Early identification of high-risk patients allows for timely intervention, such as preparing necessary medications and equipment, to improve procedural success and reduce postoperative complications. However, as a single-center retrospective study, external validation in a multicenter cohort is required to enhance the model's generalizability and clinical applicability.

## Data Availability

The original contributions presented in the study are included in the article/Supplementary Material, further inquiries can be directed to the corresponding author/s.

## References

[1] Chandrashekhar Y Alexander T Mullasari A Kumbhani DJ Alam S Alexanderson E Resource and infrastructure-appropriate management of ST-segment elevation myocardial infarction in low- and middle-income countries. Circulation. (2020) 141(24):2004–25. 10.1161/CIRCULATIONAHA.119.041297 32539609

[2] Masuda M Nakatani D Hikoso S Suna S Usami M Matsumoto S Clinical impact of ventricular tachycardia and/or fibrillation during the acute phase of acute myocardial infarction on in-hospital and 5-year mortality rates in the percutaneous coronary intervention era. Circ J. (2016) 80(7):1539–47. 10.1253/circj.CJ-16-0183 27238618

[3] Noaman S Vogrin S Dinh D Lefkovits J Brennan AL Reid CM Percutaneous coronary intervention volume and cardiac surgery availability effect on acute coronary syndrome-related cardiogenic shock. JACC Cardiovasc Interv. (2022) 15(8):876–86. 10.1016/j.jcin.2022.01.283 35450687

[4] de Waha S Patel MR Granger CB Ohman EM Maehara A Eitel I Relationship between microvascular obstruction and adverse events following primary percutaneous coronary intervention for ST-segment elevation myocardial infarction: an individual patient data pooled analysis from seven randomized trials. Eur Heart J. (2017) 38(47):3502–10. 10.1093/eurheartj/ehx414 29020248

[5] Deo R Albert CM. Epidemiology and genetics of sudden cardiac death. Circulation. (2012) 125(4):620–37. 10.1161/CIRCULATIONAHA.111.023838 22294707 PMC3399522

[6] Ito H Maruyama A Iwakura K Takiuchi S Masuyama T Hori M Clinical implications of the ‘no reflow’ phenomenon. A predictor of complications and left ventricular remodeling in reperfused anterior wall myocardial infarction. Circulation. (1996) 93(2):223–8. 10.1161/01.CIR.93.2.223 8548892

[7] Mehta RH Starr AZ Lopes RD Hochman JS Widimsky P Pieper KS Incidence of and outcomes associated with ventricular tachycardia or fibrillation in patients undergoing primary percutaneous coronary intervention. JAMA. (2009) 301(17):1779–89. 10.1001/jama.2009.600 19417195

[8] Niccoli G Burzotta F Galiuto L Crea F. Myocardial no-reflow in humans. J Am Coll Cardiol. (2009) 54(4):281–92. 10.1016/j.jacc.2009.03.054 19608025

[9] Resnic FS Wainstein M Lee MK Behrendt D Wainstein RV Ohno-Machado L No-reflow is an independent predictor of death and myocardial infarction after percutaneous coronary intervention. Am Heart J. (2003) 145(1):42–6. 10.1067/mhj.2003.36 12514653

[10] Samsky MD Morrow DA Proudfoot AG Hochman JS Thiele H Rao SV. Cardiogenic shock after acute myocardial infarction a review. JAMA. (2021) 326(18):1840–50. 10.1001/jama.2021.18323 34751704 PMC9661446

[11] Tasar O Karabay AK Oduncu V Kirma C. Predictors and outcomes of no-reflow phenomenon in patients with acute ST-segment elevation myocardial infarction undergoing primary percutaneous coronary intervention. Coron Artery Dis. (2019) 30(4):270–6. 10.1097/MCA.0000000000000726 31026233

[12] Wang JW Chen YD Wang CH Yang XC Zhu XL Zhou ZQ. Development and validation of a clinical risk score predicting the no-reflow phenomenon in patients treated with primary percutaneous coronary intervention for ST-segment elevation myocardial infarction. Cardiology. (2013) 124(3):153–60. 10.1159/000346386 23485798

[13] Wongthida T Lumkul L Patumanond J Wongtheptian W Piyayotai D Phinyo P. Development of a clinical risk score for prediction of life-threatening arrhythmia events in patients with ST elevated acute coronary syndrome after primary percutaneous coronary intervention. Int J Environ Res Public Health. (2022) 19(4):1997. 10.3390/ijerph19041997 35206186 PMC8872110

[14] Antman EM Cohen M Bernink PJ McCabe CH Horacek T Papuchis G The TIMI risk score for unstable angina/non-ST elevation MI: a method for prognostication and therapeutic decision making. JAMA. (2000) 284(7):835–42. 10.1001/jama.284.7.835 10938172

[15] Eagle KA Lim MJ Dabbous OH Pieper KS Goldberg RJ Van de Werf F A validated prediction model for all forms of acute coronary syndrome: estimating the risk of 6-month postdischarge death in an international registry. JAMA. (2004) 291(22):2727–33. 10.1001/jama.291.22.2727 15187054

[16] Ridker PM. How common is residual inflammatory risk? Circ Res. (2017) 120(4):617–9. 10.1161/CIRCRESAHA.116.310527 28209792

[17] Libby P Nahrendorf M Swirski FK. Leukocytes link local and systemic inflammation in ischemic cardiovascular disease: an expanded “cardiovascular Continuum”. J Am Coll Cardiol. (2016) 67(9):1091–103. 10.1016/j.jacc.2015.12.048 26940931 PMC4779182

[18] Peet C Ivetic A Bromage DI Shah AM. Cardiac monocytes and macrophages after myocardial infarction. Cardiovasc Res. (2020) 116(6):1101–12. 10.1093/cvr/cvz336 31841135 PMC7177720

[19] Chen Y Li X Lin X Liang H Liu X Zhang X Complement C5a induces the generation of neutrophil extracellular traps by inhibiting mitochondrial STAT3 to promote the development of arterial thrombosis. Thromb J. (2022) 20(1):24. 10.1186/s12959-022-00384-0 35488279 PMC9051782

[20] Zhang Q Hu M Sun J Ma S. The combination of neutrophil-to-lymphocyte ratio and platelet correlation parameters in predicting the no-reflow phenomenon after primary percutaneous coronary intervention in patients with ST-segment elevation myocardial infarction. Scand Cardiovasc J. (2020) 54(6):352–7. 10.1080/14017431.2020.1783457 32597237

[21] Esenboga K Kurtul A Yamanturk YY Tan TS Tutar DE. Systemic immune-inflammation index predicts no-reflow phenomenon after primary percutaneous coronary intervention. Acta Cardiol. (2022) 77(1):59–65. 10.1080/00015385.2021.1884786 33612077

[22] Schweitzer P Keller S. The role of the initial 12-lead ECG in risk stratification of patients with acute coronary syndrome. Bratisl Lek Listy. (2001) 102(9):406–11. 11763676

[23] El-Menyar A Zubaid M AlMahmeed W Sulaiman K AlNabti A Singh R Killip classification in patients with acute coronary syndrome: insight from a multicenter registry. Am J Emerg Med. (2012) 30(1):97–103. 10.1016/j.ajem.2010.10.011 21159479

[24] Hashmi KA Adnan F Ahmed O Yaqeen SR Ali J Irfan M Risk assessment of patients after ST-segment elevation myocardial infarction by Killip classification: an institutional experience. Cureus. (2020) 12(12):e12209. 10.7759/cureus.12209 33489617 PMC7815264

[25] Liu Y Ye T Chen K Wu G Xia Y Wang X A nomogram risk prediction model for no-reflow after primary percutaneous coronary intervention based on rapidly accessible patient data among patients with ST-segment elevation myocardial infarction and its relationship with prognosis. Front Cardiovasc Med. (2022) 9:966299. 10.3389/fcvm.2022.966299 36003914 PMC9393359

[26] Van de Werf F Janssens L Brzostek T Mortelmans L Wackers FJ Willems GM Short-term effects of early intravenous treatment with a beta-adrenergic blocking agent or a specific bradycardiac agent in patients with acute myocardial infarction receiving thrombolytic therapy. J Am Coll Cardiol. (1993) 22(2):407–16. 10.1016/0735-1097(93)90044-2 8335810

[27] Clemente-Moragón A Gómez M Villena-Gutiérrez R Lalama DV García-Prieto J Martínez F Metoprolol exerts a non-class effect against ischaemia-reperfusion injury by abrogating exacerbated inflammation. Eur Heart J. (2020) 41(46):4425–40. 10.1093/eurheartj/ehaa733 33026079 PMC7752252

[28] Sathnur N Ebin E Benditt DG. Sinus node dysfunction. Cardiol Clin. (2023) 41(3):349–67. 10.1016/j.ccl.2023.03.013 37321686

[29] Scarpone M Cenko E Manfrini O. Coronary no-reflow phenomenon in clinical practice. Curr Pharm Des. (2018) 24(25):2927–33. 10.2174/1381612824666180702112536 29962336

[30] Yu Y Yang BP. Sodium nitroprusside injection immediately before balloon inflation during percutaneous coronary intervention. World J Clin Cases. (2021) 9(36):11248–54. 10.12998/wjcc.v9.i36.11248 35071555 PMC8717507

[31] Bai M Lu A Pan C Hu S Qu W Zhao J Veno-arterial extracorporeal membrane oxygenation in elective high-risk percutaneous coronary interventions. Front Med. (2022) 9:913403. 10.3389/fmed.2022.913403 PMC9178105 35692539

[32] Banning AS Adriaenssens T Berry C Bogaerts K Erglis A Distelmaier K Veno-arterial extracorporeal membrane oxygenation (ECMO) in patients with cardiogenic shock: rationale and design of the randomised, multicentre, open-label EURO SHOCK trial. EuroIntervention. (2021) 16(15):e1227–e36. 10.4244/EIJ-D-20-01076 33106225 PMC9725005

