# Supplementary material for: A nomogram for predicting intraoperative risk during primary percutaneous coronary intervention based on rapidly obtained data from ST-segment elevation myocardial infarction patients
Source: Front Cardiovasc Med. 2026 Feb 12;13:1691709. doi: 10.3389/fcvm.2026.1691709 (PMC12936023; doi:10.3389/fcvm.2026.1691709)
Supplement: Supplementary file 1 [file Table1.docx]

**
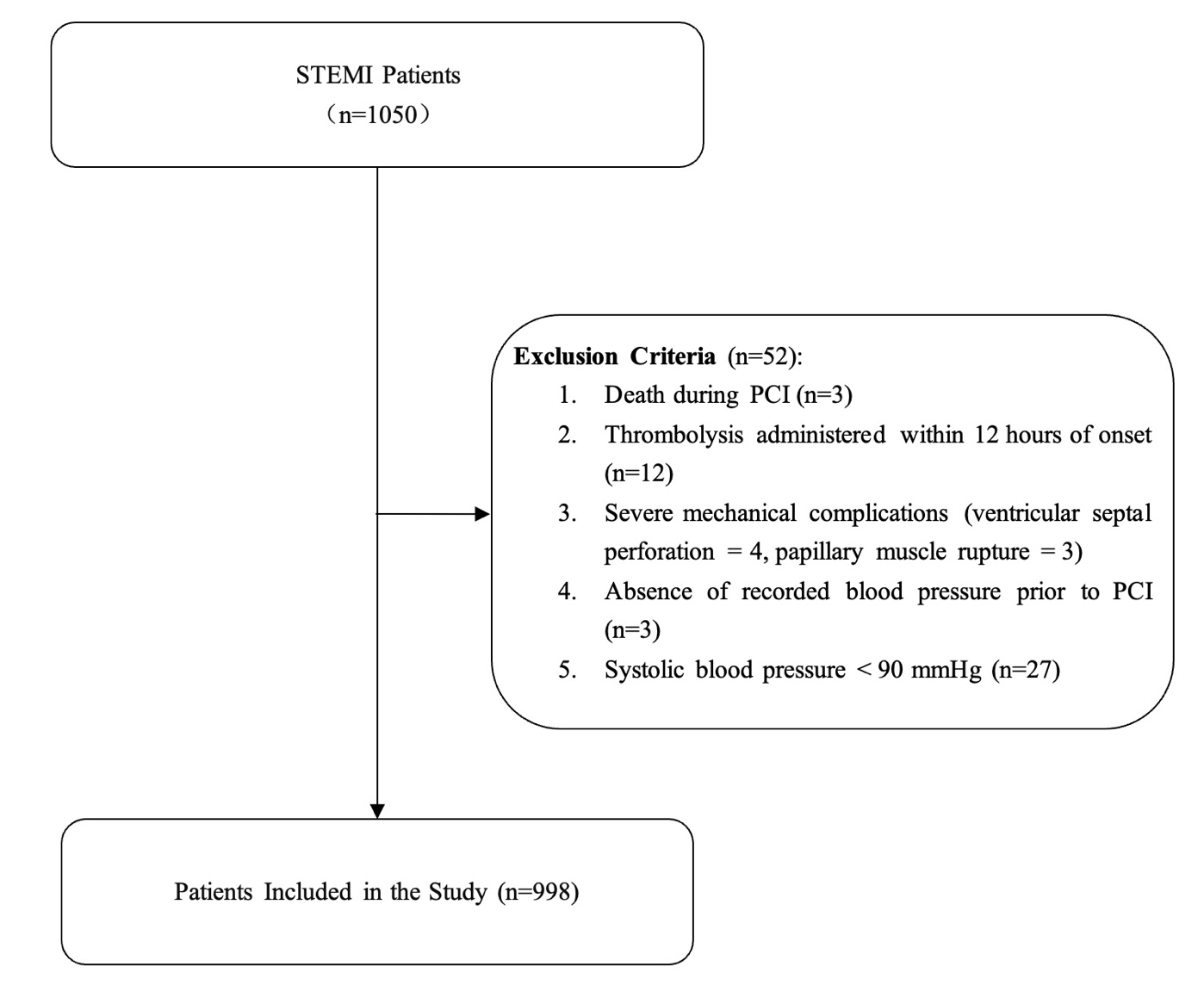
**

Figure S1. Flowchart of patient selection. Legend: A total of 1050 STEMI patients undergoing emergency PCI were screened. After applying exclusion criteria, 998 patients were included in the final analysis.


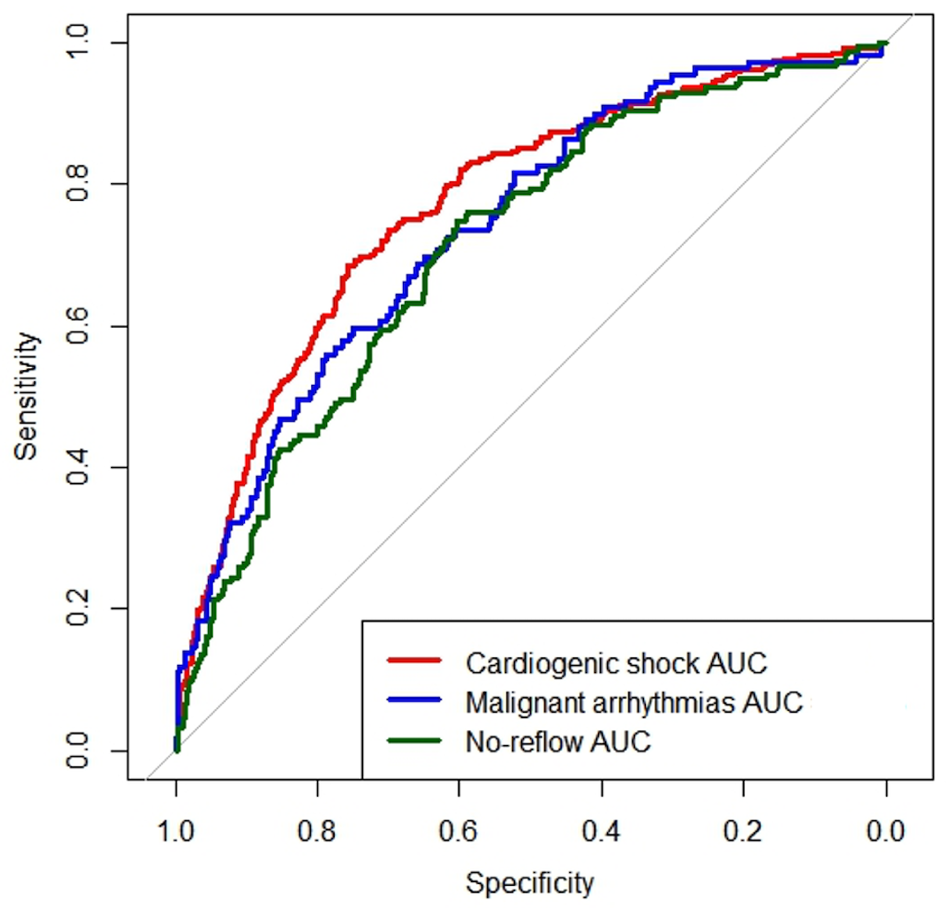


Figure S2. Receiver operating characteristic (ROC) curves of the established model for individual clinically relevant outcomes.


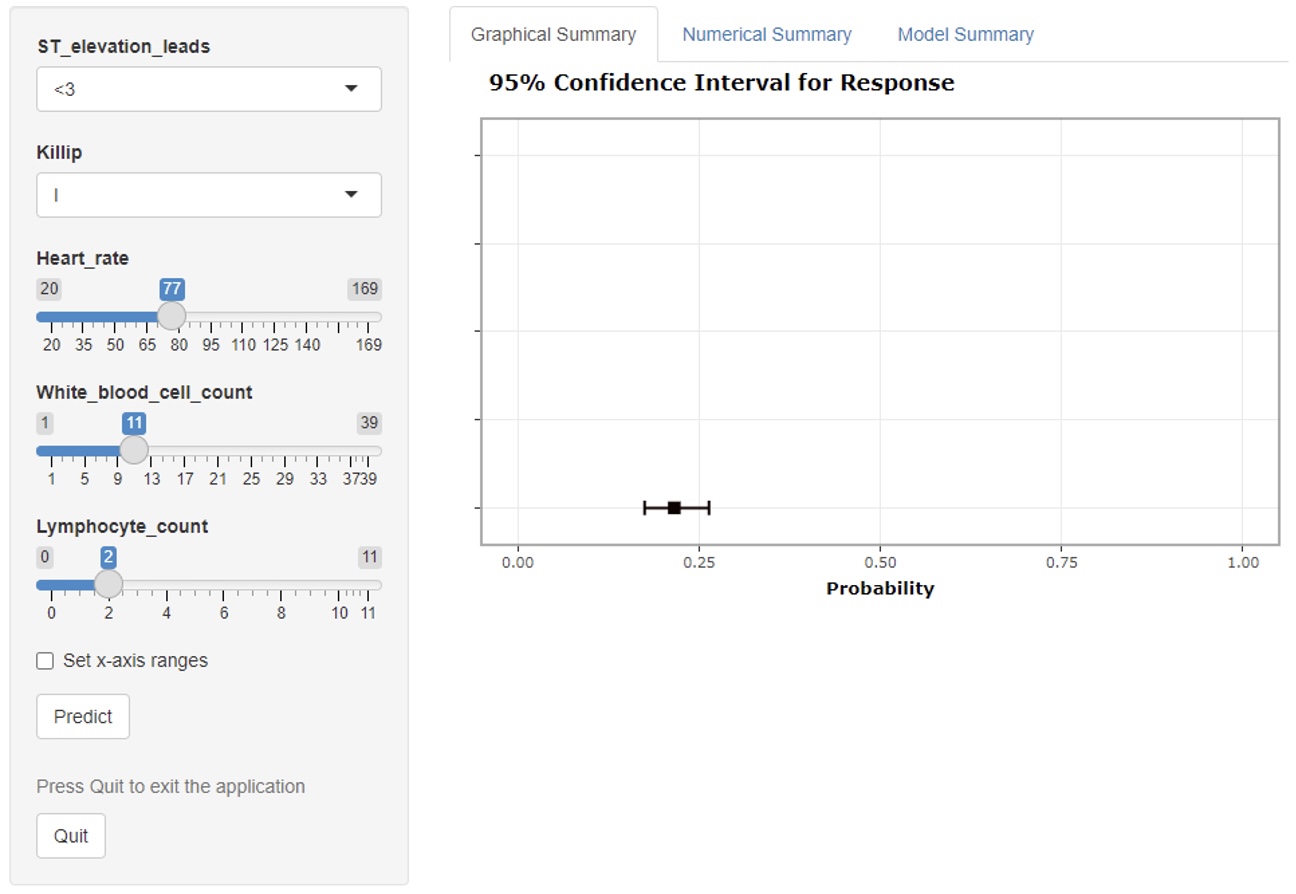


Figure S3. Online risk calculator interface. Legend: The online calculator (lxb86.shinyapps.io/dn20240324/) enables rapid intraoperative MACE risk assessment during emergency PCI using patient data.

Table S1. Baseline characteristics of participants who did and did not experience intraoperative MACE during PCI in the training set

|  |  | No-MACE  N=438 | MACE  N=259 | Statistics | P-VALUE |
| --- | --- | --- | --- | --- | --- |
| Age, Median (Q1, Q3) |  | 64.00 [55.00, 71.00] | 65.00 [55.00, 71.00] | Z = -0.419 | 0.675 |
| Sex (%) | Female | 98 (22.4) | 75 (29.0) | χ² = 3.435 | 0.064 |
|  | Male | 340 (77.6) | 184 (71.0) |  |  |
| Systolic Blood Pressure,  Median (Q1, Q3) |  | 134.50 [120.00, 150.00] | 128.00 [110.50, 148.50] | Z = -2.569 | 0.010 |
| Diastolic Blood Pressure,  Median (Q1, Q3) |  | 80.00 [70.00, 90.00] | 78.00 [70.00, 90.00] | Z = -1.993 | 0.046 |
| Red Blood Cell Count, Median (Q1, Q3) |  | 4.44 [4.09, 4.87] | 4.47 [3.97, 4.79] | Z = -0.916 | 0.360 |
| Hemoglobin, Median (Q1, Q3) |  | 135.00 [125.00, 147.75] | 136.00 [123.00, 146.00] | Z = -0.42 | 0.674 |
| White Blood Cell Count, Median (Q1, Q3) |  | 9.46 [7.65, 11.72] | 11.89 [9.60, 14.62] | Z = -8.636 | <0.001 |
| Mean Platelet Volume, Median (Q1, Q3) |  | 10.65 [10.00, 11.30] | 10.50 [9.80, 11.25] | Z = -1.266 | 0.206 |
| Neutrophil Count, Median (Q1, Q3) |  | 6.69 [4.87, 8.88] | 9.46 [7.63, 12.07] | Z = -10.596 | <0.001 |
| Platelet Count, Median (Q1, Q3) |  | 208.00 [173.00, 246.00] | 226.00 [190.50, 273.00] | Z = -4.248 | <0.001 |
| Lymphocyte Count, Median (Q1, Q3) |  | 1.75 [1.26, 2.47] | 1.35 [1.02, 1.81] | Z = -5.809 | <0.001 |
| Heart Rate, Median (Q1, Q3),  (beats/min) |  | 76.00 [66.00, 88.00] | 76.00 [65.00, 90.00] | Z = -0.154 | 0.878 |
| High-sensitivity Troponin, Median (Q1, Q3) |  | 0.39 [0.05, 3.28] | 0.96 [0.07, 8.86] | Z = -3.221 | 0.001 |
| Time from Chest Pain to PCI (minutes), Median (Q1, Q3) |  | 148.00 [74.25, 370.00] | 220.00 [120.00, 498.50] | Z = -4.128 | <0.001 |
| Blood Glucose, Median (Q1, Q3) |  | 7.11 [5.78, 9.84] | 7.48 [6.10, 9.95] | Z = -1.678 | 0.093 |
| Fibrinogen, Median (Q1, Q3) |  | 3.09 [2.77, 3.61] | 3.15 [2.74, 3.65] | Z = -1.033 | 0.302 |
| Na, Median (Q1, Q3) |  | 141.85 [139.10, 144.30] | 141.20 [138.90, 143.70] | Z = -1.726 | 0.084 |
| K, Median (Q1, Q3) |  | 3.86 [3.59, 4.13] | 3.91 [3.64, 4.28] | Z = -2.051 | 0.040 |
| Pulse Pressure, Median (Q1, Q3) |  | 51.00 [42.00, 60.00] | 50.00 [40.00, 60.00] | Z = -2.26 | 0.024 |
| Smoking History, n (%) | No | 272 (62.1) | 172 (66.4) | χ² = 1.127 | 0.288 |
|  | Yes | 166 (37.9) | 87 (33.6) |  |  |
| Hypertension, n (%) | No | 172 (39.3) | 115 (44.4) | χ² = 1.564 | 0.211 |
|  | Yes | 266 (60.7) | 144 (55.6) |  |  |
| Diabetes, n (%) | No | 311 (71.0) | 194 (74.9) | χ² = 1.052 | 0.305 |
|  | Yes | 127 (29.0) | 65 (25.1) |  |  |
| Killip Classification, n (%) | 1 | 362 (82.6) | 170 (65.6) | χ² = 46.405 | <0.001 |
|  | 2 | 61 (13.9) | 42 (16.2) |  |  |
|  | 3 | 15 (3.4) | 47 (18.1) |  |  |
| Hyperlipidemia, n (%) | No | 375 (85.6) | 231 (89.2) | χ² = 1.529 | 0.216 |
|  | Yes | 63 (14.4) | 28 (10.8) |  |  |
| Pre-PCI Cardiac Arrest, n (%) | 0 | 433 (98.9) | 246 (95.0) | χ² = 8.248 | 0.004 |
|  | 1 | 5 (1.1) | 13 (5.0) |  |  |
| Transferred from Another Hospital, n (%) | No | 305 (69.6) | 153 (59.1) | χ² = 7.595 | 0.006 |
|  | Yes | 133 (30.4) | 106 (40.9) |  |  |
| Prehospital Medication, n (%) | No | 343 (78.3) | 182 (70.3) | χ² = 5.236 | 0.022 |
|  | Yes | 95 (21.7) | 77 (29.7) |  |  |
| ST-segment Elevation ≥ 3 Leads, n (%) | <3 | 224 (51.1) | 70 (27.0) | χ² = 37.825 | <0.001 |
|  | ≥3 | 214 (48.9) | 189 (73.0) |  |  |
| Visible Thrombus on Coronary Angiography, n (%) | No | 150 (34.2) | 67 (25.9) |  |  |
|  | Yes | 288 (65.8) | 192 (74.1) | χ² = 4.944 | 0.026 |
| Number of Stents, n (%) | 1 | 244 (55.7) | 143 (55.2) | Fisher | 0.123 |
|  | 2 | 146 (33.3) | 81 (31.3) |  |  |
|  | 3 | 40 (9.1) | 24 (9.3) |  |  |
|  | 4 | 8 (1.8) | 7 (2.7) |  |  |
|  | 5 | 0 (0.0) | 4 (1.5) |  |  |
| IABP, n (%) | No | 429 (97.9) | 238 (91.9) | χ² = 13.047 | <0.001 |
|  | Yes | 9 (2.1) | 21 (8.1) |  |  |

IABP：Intra-Aortic Balloon Pump

Table S2. Comparison of baseline characteristics between the training set and validation set.

|  |  | Training Set | Validation Set | Statistics | P-Value |
| --- | --- | --- | --- | --- | --- |
|  |  | N=697 | N=301 |  |  |
| Age, Median (Q1, Q3) |  | 64.0 [55.0, 71.0] | 65.0 [56.0, 71.0] | Z= -1.080 | 0.369 |
| Sex (%) | Female | 173 (24.8) | 66 (21.9) | χ²= 1.505 | 0.367 |
|  | Male | 524 (75.2) | 235 (78.1) |  |  |
| Systolic Blood Pressure, Median (Q1, Q3) |  | 130.0 [118.0, 150.0] | 130.0 [112.0, 149.0] | Z= -1.642 | 0.082 |
| Diastolic Blood Pressure, Median (Q1, Q3) |  | 80.0 [70.0, 90.0] | 80.0 [70.0, 90.0] | Z= -1.015 | 0.848 |
| Red Blood Cells,  Median (Q1, Q3) |  | 4.4 [4.0, 4.8] | 4.5 [4.1, 4.8] | Z= -0.978 | 0.655 |
| Hemoglobin,  Median (Q1, Q3) |  | 136.0 [124.0, 147.0] | 137.0 [125.0, 148.0] | Z= -1.309 | 0.716 |
| White Blood Cells,  Median (Q1, Q3) |  | 10.3 [8.2, 12.8] | 10.1 [8.1, 13.0] | Z= -0.111 | 0.87 |
| Mean Platelet Volume, Median (Q1, Q3) |  | 10.6 [9.9, 11.3] | 10.5 [9.8, 11.2] | Z= -0.295 | 0.204 |
| Neutrophils,  Median (Q1, Q3) |  | 7.7 [5.6, 10.3] | 7.7 [5.5, 10.5] | Z= -0.474 | 0.818 |
| Platelets, Median (Q1, Q3) |  | 214.0 [180.0, 256.0] | 213.0 [176.0, 260.0] | Z= -0.584 | 0.67 |
| Lymphocytes,  Median (Q1, Q3) |  | 1.6 [1.1, 2.2] | 1.6 [1.1, 2.2] | Z= -0.370 | 0.454 |
| Heart Rate,  Median (Q1, Q3),  times per minute |  | 76.0 [66.0, 88.0] | 74.0 [65.0, 86.0] | Z= -0.642 | 0.033 |
| High-Sensitivity Troponin, Median (Q1, Q3) |  | 0.6 [0.0, 4.2] | 0.5 [0.1, 3.7] | Z= -0.276 | 0.983 |
| Time from Chest Pain Onset to PCI,  Median (Q1, Q3) |  | 180.0 [87.0, 414.0] | 158.0 [81.0, 325.0] | Z= -2.187 | 0.078 |
| Blood Glucose, Median (Q1, Q3) |  | 7.2 [5.8, 9.9] | 7.2 [5.7, 9.5] | Z= -0.541 | 0.646 |
| Fibrinogen, Median (Q1, Q3) |  | 3.1 [2.8, 3.6] | 3.2 [2.7, 3.7] | Z= -0.415 | 0.74 |
| Sodium, Median (Q1, Q3) |  | 141.7 [139.0, 144.0] | 141.2 [139.2, 143.6] | Z= -0.497 | 0.209 |
| Potassium,  Median (Q1, Q3) |  | 3.9 [3.6, 4.2] | 3.9 [3.6, 4.2] | Z= -0.363 | 0.131 |
| Pulse Pressure,  Median (Q1, Q3) |  | 50.0 [40.0, 60.0] | 50.0 [40.0, 60.0] | Z= -1.680 | 0.012 |
| Smoking History, n (%) | No | 444 (63.7) | 193 (64.1) | χ²= 0.24 | 0.957 |
|  | Yes | 253 (36.3) | 108 (35.9) |  |  |
| Hypertension, n (%) | No | 287 (41.2) | 141 (46.8) | χ²= 4.215 | 0.112 |
|  | Yes | 410 (58.8) | 160 (53.2) |  |  |
| Diabetes Mellitus, n(%) | No | 505 (72.5) | 228 (75.7) | χ²= 1.821 | 0.316 |
|  | Yes | 192 (27.5) | 73 (24.3) |  |  |
| Killip Classification, n(%) | 1 | 532 (76.3) | 228 (75.7) | χ²= 2.643 | 0.415 |
|  | 2 | 103 (14.8) | 39 (13.0) |  |  |
|  | 3 | 62 (8.9) | 34 (11.3) |  |  |
| Hyperlipidemia, n(%) | No | 606 (86.9) | 269 (89.4) | χ²= 1.807 | 0.335 |
|  | Yes | 91 (13.1) | 32 (10.6) |  |  |
| Cardiac Arrest Before PCI, n(%) | 0 | 679 (97.4) | 292 (97.0) | χ²= 0.199 | 0.879 |
|  | 1 | 18 (2.6) | 9 (3.0) |  |  |
| Transfer from Other Hospital, n (%) | No | 458 (65.7) | 220 (73.1) | χ²= 8.243 | 0.027 |
|  | Yes | 239 (34.3) | 81 (26.9) |  |  |
| Medication Treatment at Other Hospital, n (%) | No | 525 (75.3) | 257 (85.4) | χ²= 20.416 | 0.001 |
|  | Yes | 172 (24.7) | 44 (14.6) |  |  |
| ST-Elevation in >3 Leads,  n (%) | <3 | 294 (42.2) | 142 (47.2) | χ²= 3.264 | 0.164 |
|  | ≥3 | 403 (57.8) | 159 (52.8) |  |  |
| Visible Thrombus on Coronary Angiography,  n (%) | No | 217 (31.1) | 81 (26.9) | χ²= 2.788 | 0.207 |
|  | Yes | 480 (68.9) | 220 (73.1) |  |  |
| Number of Stents, n (%) | 1 | 387 (55.5) | 162 (53.8) | χ²= 3.525 | 0.688 |
|  | 2 | 227 (32.6) | 108 (35.9) |  |  |
|  | 3 | 64 (9.2) | 21 (7.0) |  |  |
|  | 4 | 15 (2.2) | 8 (2.7) |  |  |
|  | 5 | 4 (0.6) | 2 (0.7) |  |  |
| IABP, n (%) | No | 667 (95.7) | 287 (95.3) | χ²= 0.091 | 0.939 |
|  | Yes | 30 (4.3) | 14 (4.7) |  |  |

IABP：Intra-Aortic Balloon Pump

Table S3. Variance inflation factors of variables selected by LASSO regression.

| **Parameter** | **VIF** |
| --- | --- |
| White Blood Cell Count | 1.363631 |
| Killip Classification | 1.306956 |
| Heart Rate | 1.190473 |
| Lymphocyte Count | 1.241768 |
| ST-Elevation in Leads | 1.164839 |

Table S4. Multivariable logistic regression analysis for intraoperative MACE in the training set.

| Variables | β | S.E. | OR (95%CI) | P-Value |
| --- | --- | --- | --- | --- |
| White Blood Cell Count | 0.355 | 0.126 | 1.43(1.15-2.00) | 0.005 |
| ST-segment Elevation ≥ 3 Leads |  |  |  |  |
| No |  |  | Reference |  |
| Yes | 0.609 | 0.203 | 1.84(1.24-2.75) | 0.003 |
| Lymphocyte Count | -0.689 | 0.182 | 0.50(0.32-0.70) | <0.001 |
| Killip Classification |  |  |  |  |
| 1 |  |  | Reference |  |
| 2 | 0.313 | 0.266 | 1.37(0.808-2.29) | 0.239 |
| 3 | 1.967 | 0.396 | 7.15(3.36-15.98) | <0.001 |
| Heart Rate | -0.015 | 0.005 | 0.99(0.98-0.99) | 0.005 |
